# Facial Skin Necrosis after Facelift Surgery

**DOI:** 10.29252/wjps.9.1.106

**Published:** 2020-01

**Authors:** Ali Manafi, Mokhtar Asaadi, Navid Manafi

**Affiliations:** 1 Department of Plastic Surgery, School of Medicine, Iran University of Medical Sciences, Tehran, Iran; 2 Saint Barnabas Medical Center, Department of Plastic Surgery, Livingston, New Jersey, USA

**Keywords:** Face, Skin, Necrosis, Facelift, Surgery, Smoking

**DEAR EDITOR**

We would like to describe a catastrophic case of skin necrosis in a heavy smoker patient after facelift surgery which was managed with non-surgical wound care and conservative treatment.

Our patient is a 57-year-old smoker woman presented with bilateral extensive ulcers on the both sides of the face with sharp and distinct margins. She had undergone face lift procedure bilaterally 2 months earlier in another center. In the early postoperative period she started smoking again, which then ischemic blisters and discoloration with undermined skin happened, which was a sign of impending necrosis to occur. Eventually complete necrosis and skin loss happened, which was subsequently debrided by the surgeon 3 weeks postoperatively. Although she was seeking a rapid magic solution for her problem, the lesion was a dramatic subtotal skin loss.

She did not have any relevant past history, including rheumatologic or collagen vascular disease. The lesion was painless and had a clean red base. She was a heavy smoker 30 pack/year of 30 years of 1 packs a day of cigarette smoking. The evaluations were pointing towards the diagnosis of flap necrosis which has happened because of insufficient blood supply due to continuous cigarette smoking. 

During face lift procedures, the facial perforating arteries are frequently cut, hence the blood is only reached from subdermal plexus of the adjacent intact skin.^1^ In patients with poor oxygenation, like this case, flap circulation can be compromised and due to stagnation of blood flow and thrombosis of microcirculation of venous and arterial systems, subsequent skin necrosis may occur. Deciding about which treatment option to consider for these patients is not an easy one, as various options are available, including skin grafting, tissue expansion, flap transfer, keratinocyte graft with skin substitute coverage and healing by secondary intension, but the last one seems to be the most efficacious one in these patients, as chosen in this patient.^2^^, ^^3^

In performing the procedures which require good healing, careful attention to the history of cigarrete smoking and other medical conditions that compromise vascular supply including the vasculitides and vascular insufficiencies are important for proper wound healing. As a surgeon, it is imperative to discuss the possibility of occurrence of necrosis like this one in patients with insufficient blood supply. Direct questioning of the history of cigarrete smoking and its quantity should never be hesitated before performing these surgeries. Written consent should be obtained from the patient and to signify the possible complications that may occur postoperatively.

The patient was advised to stop smoking to help the skin to be healed by wound contraction. She decreased her smoking from more than 20 cigarettes a day to almost 3, and after 4 months the lesion shrinked in size considerably (Figure 1). Downsizing of the lesion has had a good positive reinforcement for her to stop smoking. However, after almost 4 weeks of not smoking, she reinitiated smoking but to a lesser degree, up to 5 cigarettes a day.

**Fig. 1:**
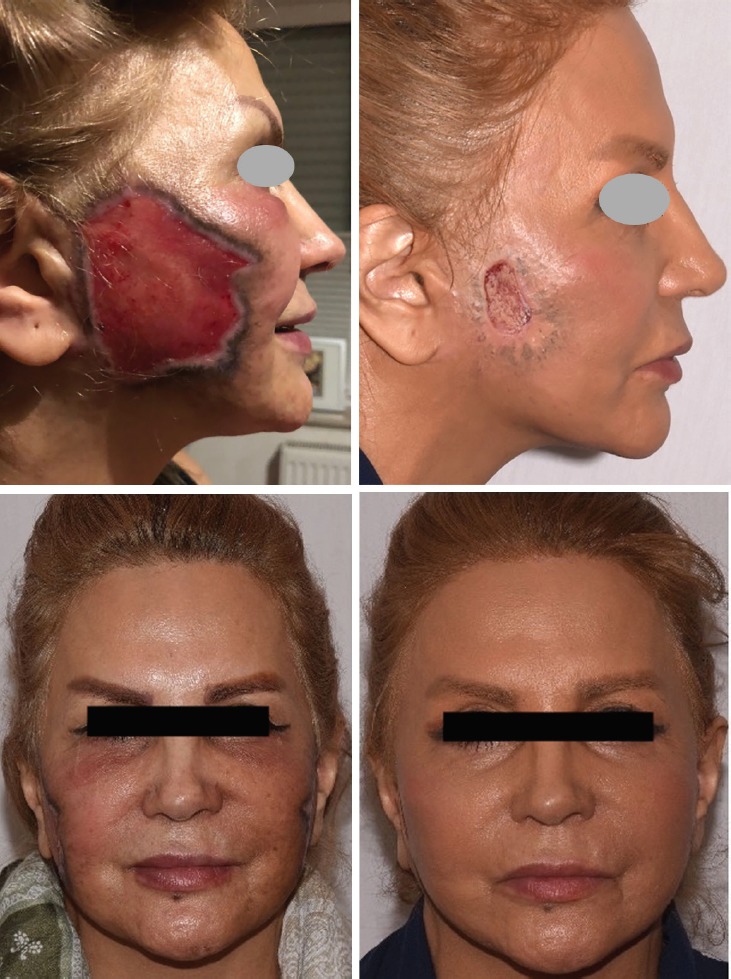
The profile and front view at presentation (left) and after 3-months of conservative management and cessation of smoking

## CONFLICT OF INTEREST

The authors declare no conflict of interest.
